# Endophytic Actinomycetes from Tea Plants (*Camellia sinensis*): Isolation, Abundance, Antimicrobial, and Plant-Growth-Promoting Activities

**DOI:** 10.1155/2018/1470305

**Published:** 2018-11-01

**Authors:** Wenna Shan, Ying Zhou, Huihui Liu, Xiaomin Yu

**Affiliations:** ^1^College of Horticulture, Fujian Agriculture and Forestry University, Fuzhou 350002, China; ^2^FAFU-UCR Joint Center for Horticultural Biology and Metabolomics, Fujian Provincial Key Laboratory of Haixia Applied Plant Systems Biology, Fujian Agriculture and Forestry University, Fuzhou 350002, China; ^3^College of Life Sciences, Fujian Agriculture and Forestry University, Fuzhou 350002, China

## Abstract

Endophytic actinomycetes are a promising source of novel metabolites with diverse biological activities. Tea plants (*Camellia sinensis*) produce arsenals of phytochemicals, which are linked to a number of medicinal and nutritional properties. However, a systematic investigation into the abundance and diversity of cultivated actinomycetes residing in tea plants has not been performed. In this study, a total of 46 actinobacteria were recovered from leaf, stem, and root samples of 15 tea cultivars collected in Fujian province, China. Their abundance and diversity were shown to be influenced by both the genotypes and tissue types of tea plants. Based on 16S RNA sequence analysis, these isolates were taxonomically grouped into 11 families and 13 genera, including* Streptomyces*,* Actinomadura*,* Kribbella*,* Nocardia*,* Kytococcus*,* Leifsonia*,* Microbacterium*,* Micromonospora*,* Mobilicoccus*,* Mycobacterium*,* Nocardiopsis*,* Piscicoccus*, and* Pseudonocardia*. The genus* Streptomyces* was most prevalent whereas rare genera,* Mobilicoccus *and* Piscicoccus*, were reported for the first time to occur as plant endophytes. PCR screening of polyketide synthase genes (PKS-I and PKS-II) and nonribosomal peptide synthetase genes (NRPS), along with antimicrobial assays against a set of bacterial and fungal pathogens, showed that endophytic actinomycetes associated with tea plants have a high potential for producing antimicrobial metabolites. Furthermore, indole acetic acid (IAA) production and 1-aminocyclopropane-1-carboxylic acid (ACC) deaminase activities were recorded in 93.5% and 21.7% of all isolates, respectively. Overall, these results indicate that endophytic actinomycetes from tea plants represent a valuable source of bioactive metabolites with antibacterial, antifungal, and plant-growth-promoting properties.

## 1. Introduction

Actinobacteria, which are characterized by high G+C DNA content and filamentous growth, constitute one of the largest bacterial phyla and are ubiquitously found in aquatic and terrestrial habitats [1]. Actinomycetes thrive in diverse ecological systems as a result of physiological versatility [2]. This group of microorganisms,* Streptomyces *in particular, are renowned for their abilities to produce a multitude of natural products with immense structural and biological diversities, many of which have applications in biotechnology, medicine, and agriculture [1, 3]. In face of global health problems such as rising diseases and widespread antibiotic resistance, there are constant calls for new antibiotics, chemotherapeutic agents, and agrochemicals. Yet in recent decades, exhausted efforts to screen soil actinomycetes for new bioactive metabolites for clinical use have met with only limited success, with repeated isolation of known compounds becoming a major issue [4]. As a result, bioprospecting actinobacteria from previously underexplored territories, such as marine sediments, hydrothermal vents, desert soils, plants, and insects, has been proposed as an important strategy to replenish the drug pipeline [4, 5].

Endophytic actinomycetes refer to actinomycetes that reside in the inner tissues of healthy host plants [6]. Endophytic strains belonging to this group have been isolated from a wide range of plants, including crop plants, medicinal plants, halophytes, and even some woody tree species [7–15]. In particular, actinomycete diversities in medicinal plants and their biotechnological applications have been the focus of several studies [16]. Accumulating evidence has shown that endophytic actinomycetes, especially those from medicinal plants, are a promising source of novel metabolites with antimicrobial, antiviral, anticancer, and anti-inflammatory properties [16–19]. They have also received much attention as biocontrol agents of plant pathogens and plant-growth promoters [20–22].

Second only to water, tea is the world's most consumed nonalcoholic beverage, with three billion kilograms being produced and consumed yearly [23]. Consumption of tea drinks is associated with a number of medicinal and nutritional benefits owing to the production of arsenals of nutraceuticals in tea plants (*Camellia sinensis*) [23, 24]. Whereas tea phytochemicals and their beneficial effects have been well characterized, studies on tea plant endophytes are rather scarce. Chemical investigations of* Pestalotiopsis *spp., fungi which are endophytic to* C. sinensis*, have led to the discovery of an array of bioactive natural products with unique structural features [25–29]. In another study, 10 butenolides were obtained from an* Aspergillus terreus* strain isolated from* C. sinensis* var.* assamica *with some showing potent anti-inflammatory activities [30]. Yan et al. discovered rubrolone B from* Streptomyces* sp. KIB-H033, an endophyte isolated from* C. sinensis*, and identified the cardioprotective activity of this compound [31]. A more recent study isolated 16 and 28 endophytic actinobacteria, respectively, from two tea cultivars* Zijuan* and* Yunkang-10* and showed that some demonstrated antimicrobial and immunomodulatory activities* in vitro* [32]. Altogether, these studies demonstrate the prevalent potential for the production of bioactive secondary metabolites among tea endophytes. Nevertheless, the diversity of endophytic actinomycetes within tea plants and their metabolic potential have not been fully explored. In this study, the abundance and diversity of cultivable actinomycetes from various tissues of different tea cultivars collected from Fujian province, China, were investigated. In addition, their antimicrobial activities against bacterial and fungal pathogens as well as their plant-growth-promoting traits were assayed.

## 2. Materials and Methods

### 2.1. Bacterial Strains, Plasmids, and Culture Conditions

*Escherichiacoli* JM109 was grown at 37°C on Luria-Bertani (LB) agar or broth supplemented with antibiotics where appropriate. Ampicillin was used for plasmid pTOPO-TA maintenance at the concentration of 100 *µ*g/ml.* Streptomyces* strains were grown at 30°C on ISP2 or ISP4 agar (Difco) or in liquid malt-yeast extract-glucose (MYG) medium (pH 7.0) containing 1% malt, 0.4% yeast extract, and 1% glucose. Other actinomycete strains were grown at 30°C on ATCC medium 172 agar or broth [33].

### 2.2. Sample Collection

Healthy young leaf, new stem, and lateral root (if available) samples with no marks or injuries were collected from tea plants (*C. sinensis*) located in seven tea plantations (25°20′ to 27°39′ N, 116°52′ to 119°34′ E) in Fujian province, China, between August 2016 and August 2017 (Table 1). From 19 tea plants, 47 tissue samples were collected. All tissue samples were placed in sterile polythene bags, brought back to the laboratory in an icebox, and subjected to processing within 24 h.

### 2.3. Isolation of Endophytic Actinomycetes

Tissue samples were washed thoroughly with running water and ultrasonicated for 10 min to remove debris. After drying, 1 g of each sample was surface-sterilized by performing a five-step procedure [7] with modifications: a 3 min wash in 70% ethanol, a 4 to 5 min wash in 8% NaOCl, a 10 min wash in 2.5% Na_2_S_2_O_3_, a 1 min wash in 70% ethanol, and a final rinse in sterile distilled water for five times. After being completely dried aseptically, samples were ground into powder with the addition of 10 mL of 0.9% NaCl and diluted 1000-fold. One hundred microliters of the dilution was plated onto eight isolation media, namely, starch-glycerol-nitrate agar (SGN), SGN supplemented with 0.5% polyvinylpyrrolidone (SGNP), tap water yeast extract agar (TWYA), TWYA modified with 0.1% tea leaf extract (TWYAPE), humic acid vitamin agar (HVA), cellulose-proline agar (CPA), xylan-arginine agar (XAA), and succinate-arginine agar (SAA), all of which were previously described [7, 34–36]. All isolation media were supplemented with 20 *µ*g/ml of nystatin to suppress fungal growth. Plates were incubated at 30°C for 2–4 weeks. Colonies displaying typical actinomycete morphologies (e.g., filamentous growth, aerial mycelium, and tough, dusty, and frequently pigmented colonies) were transferred from culture to culture to obtain clonal isolates. The pure cultures thus obtained were maintained as 7% (v/v) DMSO stock at -80°C.

To validate the efficacy of surface sterilization, a 0.1 mL aliquot of the last water wash was spread onto ISP2 media and incubated at 30°C. Only when there was no microbial growth observed on plates could the surface sterilization be considered as effective [37].

### 2.4. Molecular Identification and Phylogenetic Analysis

Pure isolates were cultured in MYG or ATCC 172 broth for 7–10 days at 30°C. Genomic DNA was extracted from pure isolates using DNeasy UltraClean Microbial Kit (QIAGEN, USA) according to the manufacture's protocol. The 16S rRNA gene was amplified by using degenerate primers (forward 16S rRNA primer 5′-GTTGGATCCAGAGTTTGATCMTGGCT-3′ and reverse 16S rRNA primer 5′- GTTGGATCCACGGYTACCTTGTTACG-3′), cloned using Zero Background pTOPO-TA Cloning Kit (Aidlab, China), and sequenced by established methods [38]. Similarity comparisons of 16S rRNA sequences and identification of phylogenetic neighbors were performed using the EzTaxon server [39]. The phylogenetic tree was constructed by the maximum likelihood method using MEGA version 7.0 with the Kimura 2-parameter model [40], taking* E. coli* as an outgroup. The robustness of the tree was evaluated by performing bootstrap analyses based on 1,000 replications [41].

### 2.5. Detection of PKS-I, PKS-II, and NRPS Biosynthetic Genes

Three sets of degenerate primers were used to detect the presence of genes encoding for polyketide synthases I and II (PKS-I and PKS-II) and nonribosomal peptide synthetases (NRPS) in recovered isolates according to the published protocols [12, 42, 43]. PKS-I gene fragments were amplified using degenerate primers KS-F (5′-CCSCAGSAGCGCSTSYTSCTSGA-3′) and KS-R (5′-GTSCCSGTSCCGTGSGYSTCSA-3′) [43]. PKS-II gene fragments were amplified using degenerate primers KS∞ (5′-TSGCSTGCTTGGAYGCSATC-3′) and KS*β* (5′-TGGAANCCGCCGAABCCTCT-3′) [12]. NRPS gene fragments were amplified using degenerate primers A3 (5′-GCSTACSYSATSTACACSTCSGG-3′) and A7R (5′-SASGTCVCCSGTSCGGTAS-3′) [42]. A negative control without DNA template was included with each set of PCR reactions.

### 2.6. Preparation of Crude Extracts for Antimicrobial Assays

Isolates which yielded positive PCR results for PKS or NRPS biosynthetic genes were subjected to antimicrobial activity tests. Seed cultures of pure isolates were grown in MYG or ATCC 172 broth for 7–10 days at 30°C. Two hundred microliters of this culture was used as inoculum for four solid media types: (1) arginine-glycerol-salt (AGS) medium [44], (2) Mannitol Soy (MS) agar medium [45], (3) ISP4 medium, and (4) ATCC 172 medium. After strain incubation for 10 days at 30°C, plates were frozen at -80°C, followed by subsequent thawing and squeezing, which allowed liquids to be liberated from agar. Extracts obtained from four media types were combined, filtered, concentrated 20-fold by rotary evaporation, and extracted with 70% methanol. Following centrifugation (12,000* g*, 10 min), the resulting supernatant was evaporated to total dryness and redissolved in water, followed by filter sterilization. Crude extracts thus obtained were stored at -20°C till subsequent use for antimicrobial assays.

### 2.7. Evaluation of Antimicrobial Activities

Antibacterial activities of isolates were evaluated by using a paper disc diffusion assay [46]. A sterile paper disc (6 mm in diameter), which was saturated with 20 *µ*L of the aforementioned crude extracts, was placed on LB agar plates inoculated with test organisms, including* Pseudomonas aeruginosa* CICC10351,* Staphylococcus aureus* CMCC(B) 26003,* Bacillus subtilis* 168, and* E. coli* BL21. Chloramphenicol (25 mg/mL, 5 *µ*L) was used as the positive control and 20 *µ*L of the methanol extract of blank media was used as the negative control. Plates were incubated at 30°C (or 37°C for* E. coli*) overnight. The diameter of inhibition zones around the paper disc was measured by caliper. Assays were carried out in triplicate.

Antifungal activities were assessed against a panel of plant pathogenic fungi, including* Magnaporthe oryzae* Guy11,* Fusarium graminearum* PH-1,* F. oxysporum* f. sp.* lycopersici*,* F. oxysporum* f. sp.* cubense*,* F*.* verticillioides *7600,* Colletotrichum* sp.,* Pestalotiopsis* sp.,* Diaporthe *sp., and* Xylaria* sp. by using the antagonism assay previously reported [35]. A fungal mycelial disc (5 mm in diameter) was removed from a potato dextrose agar (PDA) plate by a core borer and placed aseptically in the center of a fresh PDA plate. A sterile paper disc (6 mm in diameter), impregnated with 20 *µ*L of the aforementioned crude extracts, was placed approximately 2 cm apart from the center. Meanwhile, nystatin (20 mg/mL, 10 *µ*L) and 20 *µ*L of the methanol extract of blank media were included in the same plate as the positive control and the negative control, respectively. Plates were incubated at 30°C for 7–10 days. The mycelial growth of fungi was recorded. Inhibition was indicated when mycelial growth of the fungi in the direction of actinomycete crude extracts was prevented or retarded. The percentage of growth inhibition was calculated as [1-(diameter of mycelial growth in the direction of crude extract/diameter of mycelial growth in the direction of negative control)] ×100%. Assays were carried out in triplicate.

### 2.8. Quantifications of Indole Acetic Acid (IAA) Production

Production of IAA by all isolates was quantified according to Gordon and Weber [47]. Seed cultures of pure isolates were incubated in the dark in MYG broth supplemented with 5 mM tryptophan with agitation in a rotary shaker for 7–10 days at 30°C. Following centrifugation (12,000* g*, 10 min), the supernatant (0.5 mL) was mixed with 1 mL of Salkowski's reagent and incubated for another 30 min in the dark at 25°C. IAA production was quantified by measuring the absorbance at 530 nm with a spectrophotometer and comparing with the standard curve of IAA. The amount of IAA was expressed in *µ*g/mL. The experiment was performed in triplicate.

### 2.9. Screening of Bacterial Isolates for 1-Aminocyclopropane 1-Carboxylate (ACC) Deaminase Activity

Actinomycete isolates with ACC deaminase activity were screened according to their ability to use ACC as a sole source of nitrogen in the minimal medium [48]. All isolates were cultured in MYG broth for 7–10 days at 30°C. Following centrifugation (12,000* g*, 10 min), cell pellets were collected, washed twice with sterile distilled water, and resuspended in 1 mL of water. Cell suspensions were plated on agar plates containing DF salts minimal medium [49] supplemented with 3 mM ACC. The same agar plates without ACC served as the negative control and plates with 0.2% (NH_4_)_2_SO_4_ as the nitrogen source served as the positive control. Plates were incubated for 10 days at 30°C. Growth of isolates on ACC supplemented plates was compared to positive and negative controls.

### 2.10. Nucleotide Accession Numbers

The 16S rRNA sequences of the reported isolates were deposited in GenBank under the accession nos. MH432650-MH432694 and MF496983.

## 3. Results

### 3.1. Isolation of Actinomycetes from Tea Plants

A total of 15 tea cultivars were sampled from seven collection sites in Fujian province, China. These cultivars were processed into 47 tissue samples, including leaves (40.5%), stems (40.5%), and roots (19.0%). In total, 46 endophytic actinobacteria were isolated from 20 tissue samples of nine cultivars based on colony morphology and were further confirmed by 16S rRNA gene sequencing (Table 2).

**Figure 1:**
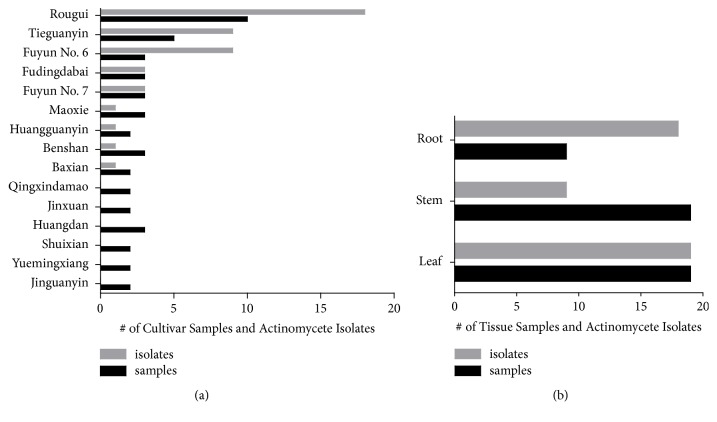
The abundance of actinobacterial isolates from different tea cultivars and tissues. (a) The abundance of actinomycete isolates recovered from different cultivars. The bar graph shows the total number of samples from each cultivar and the total number of actinomycetes isolated from each cultivar. (b) The abundance of actinomycete isolates recovered from different tissues. The bar graph shows the total number of samples from each tissue and the total number of actinomycetes isolated from each tissue.

The abundance of these isolates appeared to be influenced by tea genotypes (Figure 1(a); Supplemental Figure S1A). Despite being sampled only once, cultivar* Fuyun No. 6* yielded nine isolates from all three tissue types, with an isolate-to-sample ratio of 3:1. Cultivar* Rougui* was most frequently sampled and had an isolate-to-sample ratio of 1.8:1, the same as cultivar* Tieguanyin*. In addition, three isolates were recovered from each of two cultivars,* Fudingdabai* and* Fuyun No. 7*, showing an isolate-to-sample ratio of 1:1. These cultivars may be a good source of endophytic actinomycetes. In contrast, the remaining cultivars had either very low isolate-to-sample ratio or yielded no positive isolates (Figure 1(a)). Tissue types also had a major impact in the strain abundance. Out of 46 isolates, the maximal number of isolates was obtained from leaves (n = 19; 41.3%), followed by roots (n = 18; 39.1%) and stems (n = 9; 19.6%) (Figure 1(b); Supplemental Figure S1B). This result likely indicated the ability of endophytic actinobacteria to colonize different tissues of tea plants. Notably, although infrequently sampled compared to stems and leaves, roots yielded 18 isolates from only nine samples, with an isolate-to-sample ratio of 2:1. Leaves had an isolate-to-sample ratio of 1:1 whereas stems had a low isolate-to-sample ratio (Figure 1(b)).

The isolation efficiency of different isolation media varied greatly. The SGN medium (n = 23; 50.0%) was the most effective in the number of isolates recovered, followed by TWYAPE (n = 6; 13.0%), SGNP (n = 5; 10.9%), and CPA (n = 5; 10.9%) media. In contrary, both XAA and SAA media yielded only one isolate (Table 2).

### 3.2. Diversity of Cultivable Tea Endophytic Actinomycetes by 16S rRNA Gene Analysis

**Figure 2:**
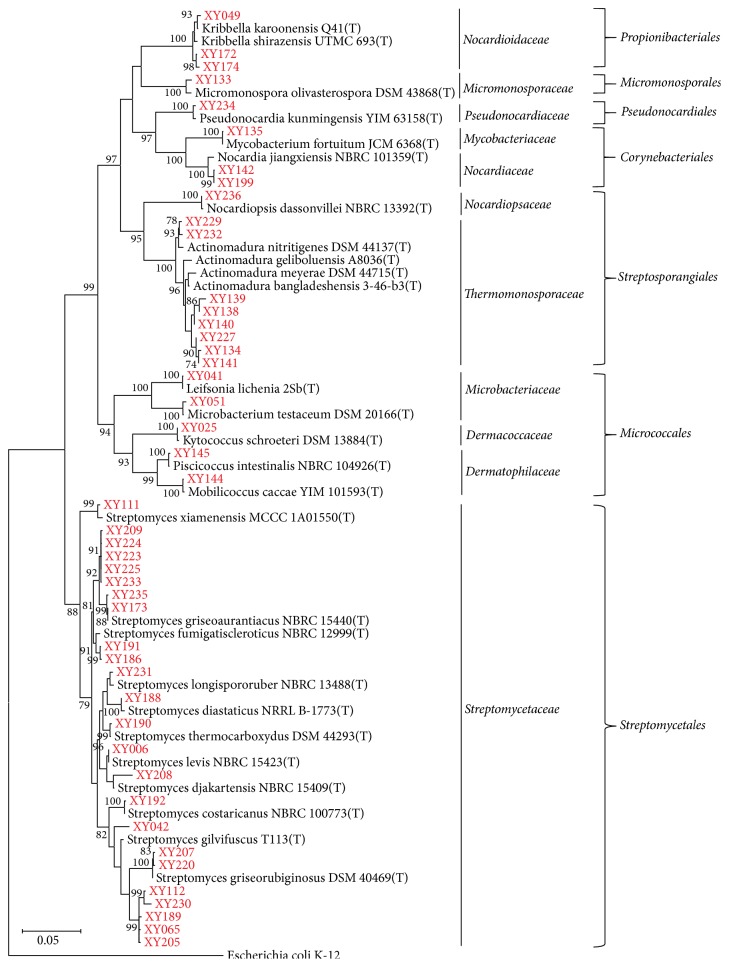
16S rRNA-based phylogenetic tree of endophytic actinobacterial isolates from tea plants, rooted with* E. coli* K-12. The alignment of nearly complete 16S rRNA sequences had 1,414 unambiguously aligned nucleotide positions. Bootstrap values greater than 70% are shown on the nodes and are based on 1,000 replicates. Sequences of closest type strains were retrieved from the EzTaxon server. The scale bar represents 0.05 substitutions per site. The isolates recovered from the current study are highlighted in red.

Analysis of 16S rRNA sequences demonstrated relatively high diversity in cultivable endophytic actinomycetes from tea plants (Figure 2). At the family level, they were distributed among 11 families: Streptomycetaceae (51.1%), Thermomonosporaceae (17.0%), Nocardioidaceae (6.4%), Dermatophilaceae (4.3%), Nocardiaceae (4.3%), Microbacteriaceae (4.3%), Nocardiopsaceae (4.3%), Pseudonocardiaceae (2.1%), Dermacoccaceae (2.1%), Micromonosporaceae (2.1%), and Mycobacteriaceae (2.1%). The most frequently isolated genus was* Streptomyces* (51.1%), occurring in seven out of 15 tea cultivars examined, followed by* Actinomadura* (17.0%),* Kribbella *(6.4%), and* Nocardia* (4.3%). The other nine genera, including* Kytococcus*,* Leifsonia*,* Microbacterium*,* Micromonospora*,* Mobilicoccus*,* Mycobacterium*,* Nocardiopsis*,* Piscicoccus*, and* Pseudonocardia*, were represented by only one isolate for each genus (Supplemental Figure S1A). Although the population of endophytic actinomycetes varied among tissues, the strain diversity within each tissue did not significantly differ (Supplemental Figure S1B), again showing the colonization potential of endophytic actinomycetes throughout the entire plant as observed in other studies [9, 10].

The 16S rRNA sequences of most isolates exhibited high levels of similarities (98.69–100%) with close type strains in the EzTaxon database (Figure 2; Supplemental Table S1). In contrast, six* Streptomyces* isolates shared lower than 98.65% sequence similarity with 16S rRNA sequences from validly described type strains and they likely represented novel species (Supplemental Table S1). Interestingly, some type strains also originated from plant endophytic environments. For example, XY041, a strain isolated from the leaf of* Tieguanyin* tea cultivar, shared 100% 16S rRNA sequence identity with* Leifsonia lichenia *2Sb isolated from lichen from Tokyo, Japan [50]. Strain XY111 isolated from the root of* Tieguanyin* shared 99.38% sequence similarity with* Streptomyces xiamenensis* MCCC 1A01550, a type strain originating from the mangrove sediment collected in Fujian, China [51]. Strain XY234, which was isolated from the stem of cultivar* Fuyun No. 6*, showed 99.35% sequence similarity to endophytic* Pseudonocardia kunmingensis* YIM 63158 isolated from the root of* Artemisia annua *from Yunnan, China [52]. These results imply that some plant-associated actinobacterial species do not have host preferences and hence are widely distributed in different plant taxa, similar to the observation in a previous study [9].

Furthermore, from the 16S rRNA phylogenetic tree, clustering of strains isolated from multiple sampling sites or from different tea cultivars was also noticed (Figure 2). For example,* Actinomadura* sp. XY134, XY138, XY139, XY140, XY141, and XY227, which were clustered into a monophyletic clade in the tree, shared high sequence similarities among themselves. They were isolated from four tea cultivars. Likewise, isolated from four tea cultivars located at five sampling sites,* Streptomyces* sp. XY189, XY065, XY205, XY230, and XY112 grouped into a single branch, which was supported by a high bootstrap value. The results indeed suggest a widespread occurrence of these isolates in the endosphere of tea plants.

### 3.3. Evaluation of Antimicrobial Activity and Screening of PKS and NRPS Genes in Selected Actinomycetes

All isolates were evaluated for their biosynthetic potential to produce secondary metabolites by PCR screening of PKS-I, PKS-II, and NRPS genes, using degenerate primers previously reported [12, 42, 43] (Supplemental Table S2). NRPS genes were detected in 32 isolates (70.0%), whereas PKS-I and PKS-II genes were detected in 28 (61.0%) and 25 (54.3%) isolates, respectively. These results likely reveal the vast potential for secondary metabolite biosynthesis among tea endophytic actinomycetes.

To select potential antagonistic isolates, isolates showing positive amplifications of PKS or NRPS genes were subjected to bioactivity screening. Antimicrobial activities of pooled extracts produced by PCR-positive isolates after growth in four different media were evaluated against a panel of microorganisms, including four bacteria and nine fungal phytopathogens. Eleven of the 37 tested strains (29.7%) showed activities against at least one of the assayed bacteria while seven strains (18.9%) were active against at least one of the fungal phytopathogens (Supplemental Tables S2 and S3). In antibacterial assays, activities against* B. subtilis *were the most common (10 isolates, 27.0%), followed by activities towards* S. aureus* (five isolates, 13.5%). None of the isolates were found to be active against* E. coli *or* P. aeruginosa*. Four isolates, XY191, XY192, XY208, and XY227, inhibited both* B. subtilis* and* S. aureus*. In particular, three isolates (XY191, XY192, and XY208) demonstrated relatively strong inhibitory effects against* S. aureus *(Supplemental Table S2). In antifungal assays, isolate XY006 exhibited a broad spectrum of activities, active against seven out of nine tested fungi (Supplemental Table S3).

### 3.4. Screening of Plant-Growth-Promoting Traits of Actinobacterial Isolates

To further explore the potential of tea endophytes for plant-growth promotion, the ability to produce plant-growth-promoting hormone IAA and ACC deaminase activity were measured for all isolates. Biosynthesis of IAA in the presence of tryptophan was found to be positive in 43 (93.5%) isolates. The quantitative production of IAA varied greatly among isolates, ranging between 2.2 and 43.1 *µ*g/mL.* Microbacterium testaceum* XY051 produced the most IAA (43.1 *µ*g/mL), followed by* Piscicoccus intestinalis* XY145 (18.7 *µ*g/mL) and* Streptomyces levis* XY006 (12.7 *µ*g/mL) (Supplemental Table S4). Ten (21.7%) isolates were tested positive for ACC deaminase activity, as indicated by their growth on DF salts minimal medium supplemented with ACC (Supplemental Table S4). In total, eight isolates (XY006, XY065, XY188, XY207, XY220, XY230, XY231, and XY236) were positive for both IAA production and ACC deaminase activity.

## 4. Discussion

Endophytes, especially endophytic actinomycetes isolated from various medicinal plants, represent an immense reservoir of novel metabolites with a wide range of biological activities [7, 11, 16, 19, 53, 54]. Although tea plants are relatively underexplored compared with other medicinal plants, increasing evidence has shown they harbor a diverse population of endophytic microorganisms with potential applications in therapeutics [25, 26, 28–32, 55] and biocontrol and plant-growth promotion [56, 57]. Nonetheless, the diversity of culturable actinomycetes within tea plants has yet to be comprehensively investigated. This has inspired us to explore tea plants to further understand the diversity of the endophytic actinomycete community and their biosynthetic potential for producing pharmaceutically and agriculturally useful compounds.

In the current study, a considerable diversity of cultivable endophytic actinobacteria was obtained from tea plants collected in Fujian province, China. A total of 46 actinomycetes belonging to 11 families and 13 genera were isolated from surface-sterilized tissues of nine of the 15 tea cultivars sampled (Table 2, Figure 2). Compared to other tea cultivars, higher strain diversity was observed in cultivars* Tieguanyin*,* Rougui*, and* Fuyun No. 6, *indeed suggesting that the actinobacterial community within plants is affected by host plant genotypes (Supplemental Figure S1).* Streptomyces *was the predominant genus (51.1%), consistent with previous studies showing* Streptomyces* as the most frequently occurring genus in other host plants [7, 9–11, 14]. Isolate XY208 shared 98.19% sequence similarity with the type strain* S. djakartensis *NBRC 15409 and it clustered singly in the phylogenetic tree. Isolates XY112, XY230, XY189, XY065, and XY205 formed a monophyletic clade in the tree which was supported by a high bootstrap value (Figure 2). The sequence similarities between these five isolates and the closest type strain* S. gilvifuscus* T113 ranged between 97.64 and 98.33%. Meanwhile, the sequence similarities among them were between 98.81 and 99.67% (Supplemental Table S1). Therefore, based on the proposed 16S rRNA sequence threshold to delineate species [58], these six* Streptomyces* isolates may represent two novel species of the genus* Streptomyces*. Nevertheless, morphological, chemotaxonomic, and phenotypic analyses are still necessary for a more thorough species assessment to confirm their novelties.

Besides* Streptomyces* sp., other genera were also recovered from tea plant tissues, including* Actinomadura, Kribbella, Nocardia, Kytococcus, Leifsonia, Microbacterium, Micromonospora, Mobilicoccus, Mycobacterium, Nocardiopsis, Piscicoccus*, and* Pseudonocardia*. Isolation of some of these genera from the plant endosphere has also been documented in a number of plants. Examples include* Actinomadura* from* Vochysia divergens* [13],* Kribbella* from* Pittosporum angustifolium* [59],* Nocardia* from* Casuarina glauca* [60],* Kytococcus* from* Rauwolfia serpentine* [10],* Micromonospora* from dandelion roots [61],* Microbacterium* and* Mycobacterium* from tea plants [32],* Nocardiopsis* from* Maytenus austroyunnanensis* [7], and* Pseudonocardia* from* Ageratum conyzoides* [62]. Noticeably, comparative analysis of the 16S rRNA gene sequences revealed that isolates XY144 and XY145, both isolated from tea roots, were closely related to* Mobilicoccus caccae* YIM 101593 and* Piscicoccus intestinalis* NBRC 104926, respectively, in the same family Dermatophilaceae (Figure 2).* M. caccae* YIM 101593 was initially isolated from the feces of a primate and* P. intestinalis* NBRC 104926 was isolated from the intestinal tract of a fish [63, 64]. To our knowledge, this is the first report on the isolation and cultivation of members in* Mobilicoccus* and* Piscicoccus* as plant endophytes. In a recent study, Wei et al. isolated 44 endophytic actinomycetes belonging to 12 genera from leaves of two tea cultivars* Zijuan* and* Yunkang-10* [32]. Members of the actinobacterial community uncovered in our study had little overlap with their study, with only* Streptomyces*,* Mycobacterium*, and* Microbacterium* being common to both studies. The discrepancies possibly suggest that the diversities of cultivable endophytic actinomycetes within tea plants may be cultivar- or geography-dependent. Indeed, cultivar-specific strain abundance and diversity have also been observed in the current study (Supplemental Figure S1A). Moreover, choices of the isolation media differed in two studies but could have a major impact on the cultivated strain diversity. Therefore, a cultivation-independent method (e.g., 16S rRNA amplicon sequencing of environmental samples) is required in the future analysis to get an unbiased estimate of the extent of actinomycete diversity in tea plants.

Actinomycetes were isolated from all tissue types including leaves, stems, and roots. Tea roots were least frequently sampled but yielded the highest isolate-to-sample ratio (Figure 1(b)), indicating the prevalence of endophytic actinomycetes in this tissue. This result is not uncommon given that roots are known as the main entry points for endophyte colonization [65]. The high incidence of actinomycetes in roots has been discovered in many other plants. For example, 54.5% of endophytic actinomycetes were isolated from the roots of 20* Azadirachta indica* trees, compared to 23.6% from the stems and 21.8% from the leaves [8]. Likewise, the highest number (52.3%) of actinomycete isolates was also recovered from the roots of several medicinal plants in India [11]. In spite of the highest occurrence of endophytic actinomycetes observed in tea roots, the strain diversity in this tissue did not appear to differ from those in leaves and stems (Supplemental Figure S1B).

To assess the biosynthetic potential of tea endophytic actinomycetes, a set of approaches were carried out, including PCR screening of secondary metabolic genes, detection of antibacterial and antifungal activities, and screening of plant-growth-promoting activities. NRPS genes could be detected from 70.0% of the isolates while the positive rates for PKS-I and PKS-II genes were 61.0% and 54.3%, respectively, revealing the high potential in producing biologically active natural products (Supplemental Table S2). We prescreened isolates by PCR targeting PKS and NRPS genes to prioritize strains for novel metabolite discovery and subsequently focused on PCR-positive isolates to test their antimicrobial activities against bacterial and fungal pathogens. Sixteen out of the 37 isolates (43.2%) exhibited antagonistic activities against at least one of the tested microorganisms, among which 11 isolates showed antibacterial activities and seven showed antifungal activities (Supplemental Tables S2 and S3). Some isolates showed antagonistic activities against multiple tested strains. For instance, isolate XY006 was active against several fungal phytopathogens. Isolate XY134 was active towards both* M. oryzae* and* B. subtilis*. Isolate XY208 was active against* B. subtilis*,* S. aureus*,* Pestalotiopsis *sp., and* Diaporthe* sp. Most interestingly, isolate XY192 demonstrated the highest activity towards both* S. aureus* and* B. subtilis*. These promising isolates warrant further chemical analysis to identify potential antimicrobial compounds, which is currently in progress.

It is noteworthy to point out that half of the isolates showing antimicrobial activities belong to* Streptomyces*, which once again confirms the reputation of this talented genus as a prolific natural product producer [66]. This finding is in accord with early reports which state that endophytic* Streptomyces* species from medicinal plants possess antimicrobial properties and thereby are potential candidates to recover novel antimicrobial natural products [11, 67]. Among the non-*Streptomyces* species,* Actinomadura* is the leading group showing antimicrobial activities (Supplemental Tables S2 and S3). This group of microorganisms has been isolated from diverse environmental samples and is noted for producing chemically and biologically diverse metabolites, such as furopyrimidine derivatives with antibacterial activities, maduropeptins with antibacterial and potent antitumor activities, and nomimicin with antimicrobial activities [68–70]. Nonetheless, the biological activities of plant-associated* Actinomadura *have rarely been explored. Given their high metabolic potential for the production of diverse metabolites, we believe it is worthwhile to mine endophytic* Actinomadura *isolates recovered in the current study as a potential source of novel bioactive compounds.

Apart from producing antimicrobial metabolites, many endophytic actinobacteria have been recognized to stimulate plant growth by a plethora of mechanisms, such as producing phytohormones, nitrogen fixation, siderophore biosynthesis, nutrient solubilization, and synthesis of ACC deaminase [12, 20]. The production of phytohormones, most notably IAA, is most frequently explored [71]. IAA is known to promote root growth and development, which in turn improves the nutrient uptake by plants [72]. Promoting plant growth by lowering plant ethylene levels through the activity of ACC deaminase is also documented in some plant endophytes [73]. As a preliminary test to understand the plant-growth-promoting potential of tea endophytes, IAA production and ACC deaminase activity were measured in all isolates. The results demonstrated that the majority of the strains (93.5%) were able to produce IAA (Supplemental Table S4). The production of this plant-growth regulator ranged between 2.2 and 43.1 *µ*g/mL in tryptophan supplemented culture medium, with the highest yield recorded in isolate XY051. This finding is in consensus with other reports which gave comparable levels of IAA production by endophytic actinomycetes isolated from other sources [20, 74, 75]. Ten out of 46 endophytic actinomycetes in the current study showed the ACC utilizing ability on the plate assay taking ACC as the sole N source and hence were positive for the ACC deaminase activity (Supplemental Table S4). Given that ACC is an important precursor for ethylene synthesis in plants, ACC deaminase-expressing microbes could reduce the ACC level and thereby alleviate the ethylene-mediated stress on plants [76]. To our interest, eight of the screened isolates demonstrated both IAA production and ACC deaminase activity. These isolates could presumably benefit the host and increase their growth. Further experimentation would be needed to explore the beneficial interactions of these isolates with plants* in vivo*. Other plant-growth-promoting properties will also be examined in the future.

## 5. Conclusion

This study is the first systematic investigation into the tissue-specific abundance, diversity, and antimicrobial and plant-growth-promoting activities of endophytic actinomycetes from tea plants. The 46 actinobacterial strains recovered are assigned to 11 families and 13 genera.* Streptomyces* isolates dominate in tea plants, with some likely representing new species. Some rare actinomycetes are also uncovered, among which* Mobilicoccus *and* Piscicoccus* sp. have not been previously reported as plant endophytes. Notably, 43.2% of the tested isolates show inhibitory activities against at least one bacterial or fungal pathogen. The positive rates for two plant-growth-promoting traits, namely, IAA production and ACC deaminase activity, are also relatively high among tea endophytic actinomycetes. Moreover, these isolates demonstrate a high metabolic potential for producing secondary metabolites. Taken together, this study reveals a high diversity of cultivable actinomycetes in tea plants, which is potentially a valuable source of bioactive metabolites with antibacterial, antifungal, and plant-growth-promoting properties.

## Figures and Tables

**Table 1:** Summary of tea sample collection.

**Location**	**Tea cultivar**	**Collection date**	**Tissue of origin**
Tea Plantation of Fujian Agriculture and Forestry University	Tieguanyin	August 30 2016	Leaf, stem, root
Tea Plantation of Fujian Agriculture and Forestry University	Rougui	August 30 2016	Leaf, stem, root
Tea Plantation of Fujian Agriculture and Forestry University	Maoxie	August 30 2016	Leaf, stem, root
Tea Plantation of Fujian Agriculture and Forestry University	Fudingdabai	August 30 2016	Leaf, stem, root
Tea Plantation of Fujian Agriculture and Forestry University	Fuyun No. 6	July 11 2017	Leaf, stem, root
Tea Plantation of Fujian Agriculture and Forestry University	Fuyun No. 7	July 11 2017	Leaf, stem, root
Tea Plantation of Fujian Agriculture and Forestry University	Benshan	July 16 2017	Leaf, stem, root
Tea Plantation of Fujian Agriculture and Forestry University	Huangdan	July 16 2017	Leaf, stem, root
Jingyan Mountain	Rougui	April 17 2017	Leaf, stem, root
Datian Tea Plantation	Rougui	July 8 2017	Leaf, stem
Datian Tea Plantation	Jinxuan	July 8 2017	Leaf, stem
Dehua Tea Plantation	Qingxindamao	July 8 2017	Leaf, stem
Shanghang Tea Plantation	Tieguanyin	October 16 2016	Leaf, stem
Fujian Tea Research Institute	Huangguanyin	April 18 2017	Leaf, stem
Fujian Tea Research Institute	Baxian	April 18 2017	Leaf, stem
Fujian Tea Research Institute	Jinguanyin	April 18 2017	Leaf, stem
Fujian Tea Research Institute	Shuixian	April 18 2017	Leaf, stem
Fujian Tea Research Institute	Yuemingxiang	April 18 2017	Leaf, stem
Wuyi Mountain	Rougui	August 5 2017	Leaf, stem

**Table 2:** Phylogenetic affiliations, isolation media, and isolation origin of endophytic actinomycetesfrom tea plants.

**Isolate**	**Isolate Identified as**	**Accession no.**	**Media name**	**Tea cultivar**	**Tissue origin**
XY006	*Streptomyces levis*	MF496983	SGN	Tieguanyin	leaf
XY025	*Kytococcus schroeteri*	MH432655	SGN	Rougui	leaf
XY041	*Leifsonia lichenia*	MH432650	TWYAPE	Tieguanyin	leaf
XY042	*Streptomyces rhizophilus*	MH432656	TWYAPE	Tieguanyin	leaf
XY049	*Kribbella karoonensis*	MH432651	TWYAPE	Tieguanyin	leaf
XY051	*Microbacterium testaceum*	MH432657	SGN	Tieguanyin	stem
XY065	*Streptomyces *sp.	MH432658	HVA	Maoxie	root
XY111	*Streptomyces xiamenensis*	MH432665	TWYA	Tieguanyin	root
XY112	*Streptomyces *sp.	MH432660	SGN	Tieguanyin	leaf
XY133	*Micromonospora olivasterospora*	MH432666	SGN	Tieguanyin	leaf
XY134	*Actinomadura geliboluensis*	MH432663	HVA	Rougui	root
XY135	*Mycobacterium fortuitum*	MH432652	SGN	Rougui	root
XY138	*Actinomadura meyerae*	MH432670	HVA	Tieguanyin	stem
XY139	*Actinomadura meyerae*	MH432661	SGN	Rougui	root
XY140	*Actinomadura bangladeshensis*	MH432662	SGN	Rougui	root
XY141	*Actinomadura geliboluensis*	MH432664	TWYAPE	Fudingdabai	root
XY142	*Nocardia jiangxiensis*	MH432653	TWYAPE	Fudingdabai	root
XY144	*Mobilicoccus caccae*	MH432659	TWYA	Rougui	root
XY145	*Piscicoccus intestinalis*	MH432654	SGN	Fudingdabai	root
XY172	*Kribbella shirazensis*	MH432669	SGN	Fuyun No. 6	root
XY173	*Streptomyces griseoaurantiacus*	MH432690	CPA	Fuyun No. 6	root
XY174	*Kribbella shirazensis*	MH432671	TWYAPE	Fuyun No. 6	root
XY186	*Streptomyces fumigatiscleroticus*	MH432688	SGN	Huangguanyin	leaf
XY188	*Streptomyces diastaticus*	MH432683	SGNP	Rougui	root
XY189	*Streptomyces* sp.	MH432684	SGNP	Rougui	root
XY190	*Streptomyces thermocarboxydus*	MH432689	SGNP	Rougui	root
XY191	*Streptomyces fumigatiscleroticus*	MH432668	SGNP	Rougui	root
XY192	*Streptomyces costaricanus*	MH432685	SGNP	Rougui	root
XY199	*Nocardia jiangxiensis*	MH432694	SAA	Fuyun No. 6	stem
XY205	*Streptomyces *sp.	MH432667	SGN	Baxian	leaf
XY207	*Streptomyces griseorubiginosus*	MH432672	SGN	Fuyun No. 6	stem
XY208	*Streptomyces *sp.	MH432673	SGN	Fuyun No. 6	leaf
XY209	*Streptomyces *sp.	MH432674	SGN	Fuyun No. 6	leaf
XY220	*Streptomyces griseorubiginosus*	MH432686	SGN	Fuyun No. 7	leaf
XY223	*Streptomyces griseoaurantiacus*	MH432687	CPA	Rougui	leaf
XY224	*Streptomyces fumigatiscleroticus*	MH432676	SGN	Fuyun No. 7	leaf
XY225	*Streptomyces griseoaurantiacus*	MH432679	CPA	Rougui	stem
XY227	*Actinomadura geliboluensis*	MH432691	SGN	Fuyun No. 6	leaf
XY229	*Actinomadura nitritigenes*	MH432680	SGN	Benshan	stem
XY230	*Streptomyces *sp.	MH432675	CPA	Rougui	stem
XY231	*Streptomyces longispororuber*	MH432677	SGN	Rougui	leaf
XY232	*Actinomadura nitritigenes*	MH432681	SGN	Rougui	leaf
XY233	*Streptomyces griseoaurantiacus*	MH432678	SGN	Rougui	leaf
XY234	*Pseudonocardia kunmingensis*	MH432692	XAA	Fuyun No. 6	stem
XY235	*Streptomyces griseoaurantiacus*	MH432682	CPA	Rougui	stem
XY236	*Nocardiopsis dassonvillei*	MH432693	SGN	Fuyun No. 7	leaf

## Data Availability

The data used to support the findings of this study are available from the corresponding author upon request.

## Supplementary Materials

Supplementary Materials Supplemental Figure S1: abundance and diversity of culturable endophytic actinomycetes from tea plants: (A) abundance and diversity of actinomycete isolates among different tea cultivars and (B) abundance and diversity of actinomycete isolates among different tea plant tissues. Supplemental Table S1: 16S rRNA sequence similarity with type strains. Supplemental Table S2: biosynthetic genes (PKS-I, PKS-II, and NRPS) and antibacterial activities of culturable endophytic actinomycetes from tea plants. Supplemental Table S3: antifungal activities of culturable endophytic actinomycetes from tea plants. Supplemental Table S4: IAA production and ACC deaminase activity of endophytic actinomycetes from tea plants.
