# Supplementary material for: Endophytic Actinomycetes from Tea Plants (Camellia sinensis): Isolation, Abundance, Antimicrobial, and Plant-Growth-Promoting Activities
Source: Biomed Res Int. 2018 Nov 1;2018:1470305. doi: 10.1155/2018/1470305 (PMC6241348; doi:10.1155/2018/1470305)
Supplement: Supplementary Materials — Supplemental Figure S1: abundance and diversity of culturable endophytic actinomycetes from tea plants: (A) abundance and diversity of actinomycete isolates among different tea cultivars and (B) abundance and diversity of actinomycete isolates among different tea plant tissues. Supplemental Table S1: 16S rRNA sequence similarity with type strains. Supplemental Table S2: biosynthetic genes (PKS-I, PKS-II, and NRPS) and antibacterial activities of culturable endophytic actinomycetes from tea plants. Supplemental Table S3: antifungal activities of culturable endophytic actinomycetes from tea plants. Supplemental Table S4: IAA production and ACC deaminase activity of endophytic actinomycetes from tea plants. [file 1470305.f1.pdf]

**Supplemental Materials**

# Endophytic actinomycetes from tea plants (*Camellia sinensis*): Isolation, abundance, antimicrobial and plant growth-promoting activities

Wenna Shan[1,2]#, Ying Zhou[2,3]#, Huihui Liu[1,2], Xiaomin Yu[2]*

1. College of Horticulture, Fujian Agriculture and Forestry University, Fuzhou 350002, China
2. FAFU-UCR Joint Center for Horticultural Biology and Metabolomics, Fujian Provincial Key Laboratory of Haixia Applied Plant Systems Biology, Fujian Agriculture and Forestry University, Fuzhou 350002, China
3. College of Life Sciences, Fujian Agriculture and Forestry University, Fuzhou 350002, China

[#] S.W. and Z.Y. contributed equally to this work.

*__Correspondence:__

Dr. Xiaomin Yu

xmyu0616@fafu.edu.cn

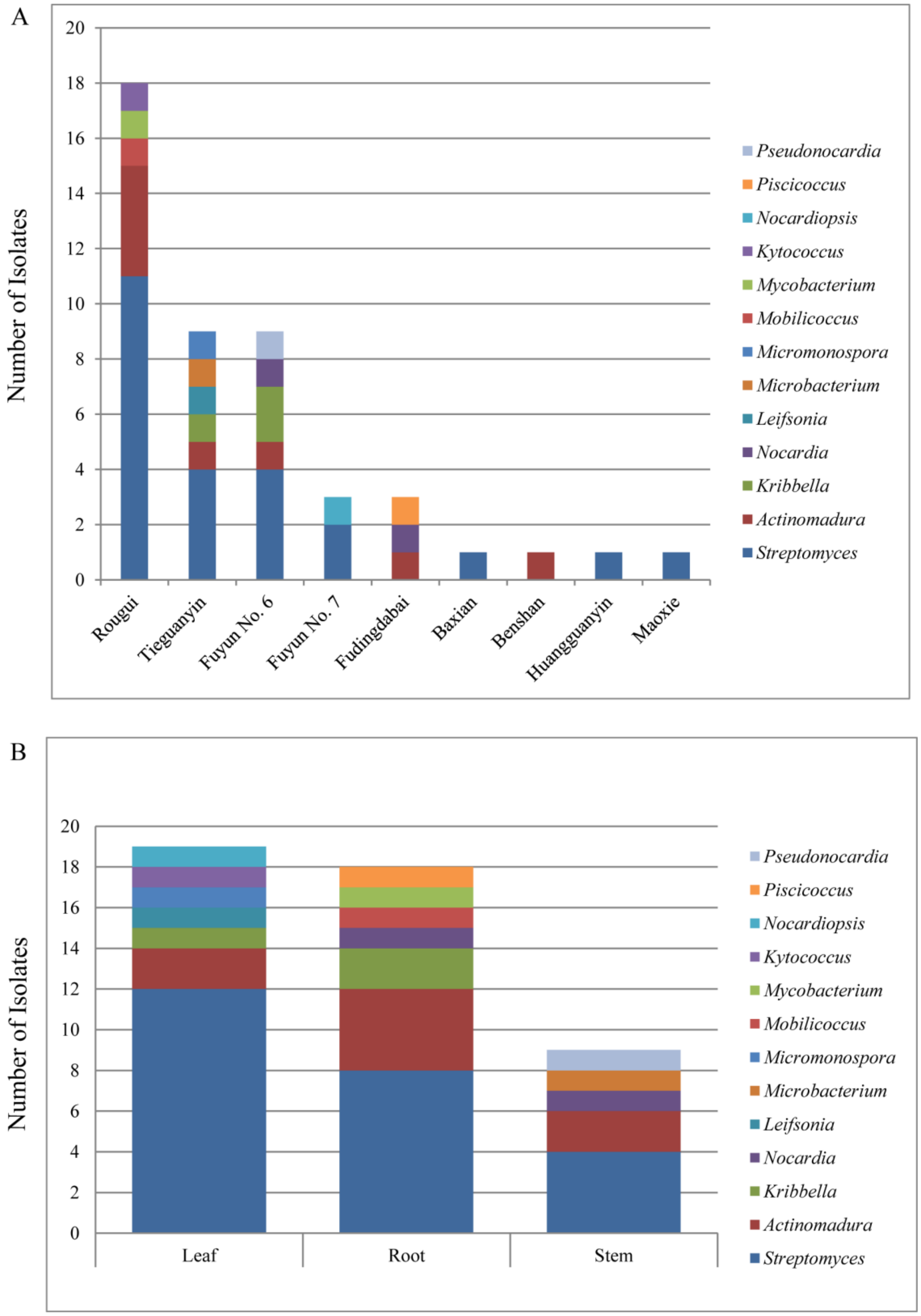


**Supplemental Figure S1.** Abundance and diversity of culturable endophytic actinomycetes from tea plants. (**A**) Abundance and diversity of actinomycete isolates among different tea cultivars. (**B**) Abundance and diversity of actinomycete isolates among different tea plant tissues.

**Supplemental Table S1. 16S rRNA sequence similarity with type strains**

| Strain Name | Accession No. | Top-hit strain | Similarity (%) |
|---|---|---|---|
| XY006 | MF496983 | *Streptomyces levis* NBRC 15423(T) | 100.00 |
| XY025 | MH432655 | *Kytococcus schroeteri* DSM 13884(T) | 99.86 |
| XY041 | MH432650 | *Leifsonia lichenia* 2Sb(T) | 100.00 |
| XY042 | MH432656 | *Streptomyces rhizophilus* JR-41(T) | 99.72 |
| XY049 | MH432651 | *Kribbella karoonensis* Q41(T) | 99.52 |
| XY051 | MH432657 | *Microbacterium testaceum* DSM 20166(T) | 99.65 |
| XY065 | MH432658 | *Streptomyces gilvifuscus* T113(T) | 98.33 |
| XY111 | MH432665 | *Streptomyces xiamenensis* MCCC 1A01550(T) | 99.38 |
| XY112 | MH432660 | *Streptomyces gilvifuscus* T113(T) | 98.05 |
| XY133 | MH432666 | *Micromonospora olivasterospora* DSM 43868(T) | 99.10 |
| XY134 | MH432663 | *Actinomadura geliboluensis* A8036(T) | 98.75 |
| XY135 | MH432652 | *Mycobacterium fortuitum* JCM 6368(T) | 99.93 |
| XY138 | MH432670 | *Actinomadura meyerae* DSM 44715(T) | 99.10 |
| XY139 | MH432661 | *Actinomadura meyerae* DSM 44715(T) | 98.82 |
| XY140 | MH432662 | *Actinomadura bangladeshensis* 3-46-b3(T) | 99.17 |
| XY141 | MH432664 | *Actinomadura geliboluensis* A8036(T) | 98.75 |
| XY142 | MH432653 | *Nocardia jiangxiensis* NBRC 101359(T) | 98.96 |
| XY144 | MH432659 | *Mobilicoccus caccae* YIM 101593(T) | 99.72 |
| XY145 | MH432654 | *Piscicoccus intestinalis* NBRC 104926(T) | 99.86 |
| XY172 | MH432669 | *Kribbella shirazensis* UTMC 693(T) | 99.29 |
| XY173 | MH432690 | *Streptomyces griseoaurantiacus* NBRC 15440(T) | 100.00 |
| XY174 | MH432671 | *Kribbella shirazensis* UTMC 693(T) | 99.07 |
| XY186 | MH432688 | *Streptomyces fumigatiscleroticus* NBRC 12999(T) | 99.17 |
| XY188 | MH432683 | *Streptomyces diastaticus* NRRL B-1773(T) | 99.72 |
| XY189 | MH432684 | *Streptomyces gilvifuscus* T113(T) | 98.26 |
| XY190 | MH432689 | *Streptomyces thermocarboxydus* DSM 44293(T) | 99.79 |
| XY191 | MH432668 | *Streptomyces fumigatiscleroticus* NBRC 12999(T) | 99.24 |
| XY192 | MH432685 | *Streptomyces costaricanus* NBRC 100773(T) | 99.79 |
| XY199 | MH432694 | *Nocardia jiangxiensis* NBRC 101359(T) | 99.03 |
| XY205 | MH432667 | *Streptomyces gilvifuscus* T113(T) | 98.33 |
| XY207 | MH432672 | *Streptomyces griseorubiginosus* DSM 40469(T) | 99.79 |
| XY208 | MH432673 | *Streptomyces djakartensis* NBRC 15409(T) | 98.19 |
| XY209 | MH432674 | *Streptomyces griseoaurantiacus* NBRC 15440(T) | 98.69 |

| | | | |
|---|---|---|---|
| XY220 | MH432686 | *Streptomyces griseorubiginosus* DSM 40469(T) | 99.79 |
| XY223 | MH432687 | *Streptomyces griseoaurantiacus* NBRC 15440(T) | 98.75 |
| XY224 | MH432676 | *Streptomyces fumigatiscleroticus* NBRC 12999(T) | 98.75 |
| XY225 | MH432679 | *Streptomyces griseoaurantiacus* NBRC 15440(T) | 98.82 |
| XY227 | MH432691 | *Actinomadura geliboluensis* A8036(T) | 98.96 |
| XY229 | MH432680 | *Actinomadura nitritigenes* DSM 44137(T) | 99.30 |
| XY230 | MH432675 | *Streptomyces gilvifuscus* T113(T) | 97.64 |
| XY231 | MH432677 | *Streptomyces longispororuber* NBRC 13488(T) | 99.51 |
| XY232 | MH432681 | *Actinomadura nitritigenes* DSM 44137(T) | 99.23 |
| XY233 | MH432678 | *Streptomyces griseoaurantiacus* NBRC 15440(T) | 98.75 |
| XY234 | MH432692 | *Pseudonocardia kunmingensis* YIM 63158(T) | 99.35 |
| XY235 | MH432682 | *Streptomyces griseoaurantiacus* NBRC 15440(T) | 99.79 |
| XY236 | MH432693 | *Nocardiopsis dassonvillei* NBRC 13392(T) | 99.86 |

**Supplemental Table S2. Biosynthetic genes (PKS-I, PKS-II and NRPS) and antibacterial activities of culturable endophytic actinomycetes from tea plants**

| Isolate no. | Antibacterial activities (zone of inhibition in mm)[a] | | | | Biosynthetic genes[b] | | |
|---|---|---|---|---|---|---|---|
| | *P. aeruginosa* | *S. aureus* | *E. coli* | *B. subtilis* | PKS-I | PKS-II | NRPS |
| XY006 | - | - | - | - | + | - | + |
| XY025 | ND | ND | ND | ND | - | - | - |
| XY041 | ND | ND | ND | ND | - | - | - |
| XY042 | - | - | - | - | + | + | + |
| XY049 | ND | ND | ND | ND | - | - | - |
| XY051 | ND | ND | ND | ND | - | - | - |
| XY065 | - | - | - | - | - | + | - |
| XY111 | - | - | - | - | - | + | + |
| XY112 | - | - | - | - | - | + | + |
| XY133 | - | - | - | - | - | + | + |
| XY134 | - | - | - | ++ (8.7±0.6) | + | - | + |
| XY135 | - | - | - | - | + | - | + |
| XY138 | - | - | - | - | + | - | + |
| XY139 | - | - | - | - | + | - | + |
| XY140 | - | - | - | ++ (9.3±0.6) | + | - | + |
| XY141 | - | - | - | - | - | - | + |
| XY142 | - | - | - | - | + | - | + |
| XY144 | ND | ND | ND | ND | - | - | - |
| XY145 | - | - | - | - | - | - | + |
| XY172 | ND | ND | ND | ND | - | - | - |
| XY173 | - | - | - | - | + | + | - |
| XY174 | ND | ND | ND | ND | - | - | - |
| XY186 | ND | ND | ND | ND | - | - | - |
| XY188 | - | - | - | - | + | + | + |
| XY189 | - | - | - | - | + | + | - |
| XY190 | - | - | - | - | + | + | - |
| XY191 | - | +++ (14.0±0.6) | - | +++ (14.0±1.0) | + | + | + |
| XY192 | - | +++ (19.3±0.6) | - | +++ (19.0±1.0) | + | + | + |
| XY199 | - | - | - | - | + | - | + |
| XY205 | ND | ND | ND | ND | - | - | - |
| XY207 | - | - | - | - | + | + | + |
| XY208 | - | +++ (12.7±0.6) | - | +++ (12.0±1.0) | + | + | + |
| XY209 | - | - | - | - | + | + | + |
| XY220 | - | - | - | - | - | + | + |
| XY223 | - | - | - | ++ (7.7±0.6) | + | + | + |
| XY224 | - | - | - | ++ (9.7±0.6) | + | + | + |
| XY225 | - | - | - | - | + | + | + |

| | | | | | | | |
|---|---|---|---|---|---|---|---|
| XY227 | - | ++ (8.7±1.2) | - | +++ (11.0±1.0) | + | - | + |
| XY229 | - | ++ (9.3±0.6) | - | - | + | + | + |
| XY230 | - | - | - | - | - | + | + |
| XY231 | - | - | - | - | + | + | + |
| XY232 | - | - | - | +++ (11.0±1.0) | + | + | + |
| XY233 | - | - | - | - | + | + | + |
| XY234 | - | - | - | ++ (8.7±0.6) | - | - | + |
| XY235 | - | - | - | - | + | + | + |
| XY236 | - | - | - | - | + | + | - |

[a] Determined by measuring the size of inhibition zone. Symbols: -, no activity; +, weak activity, inhibition zone between 0-4.9 mm; ++, moderate activity, inhibition zone between 5.0-9.9 mm; +++, strong activity, inhibition zone >10 mm. ND, not determined. Experiments were performed in triplicates. Numbers in the parenthesis indicate the size of inhibition zone in mm, which are expressed as mean ± SD.

[b] +, present; -, absent.

**Supplemental Table S3. Antifungal activities of culturable endophytic actinomycetes from tea plants**

| Isolate no. | Antifungal activities (GI%)[a] | | | | | | | | |
|---|---|---|---|---|---|---|---|---|---|
| | *M. oryzae* | *F. graminearum* | *F. oxysporum* f. sp *lycopersici* | *F. oxysporum* f. sp *cubense* | *F. verticillioides* | *Colletotrichum* sp. | *Pestalotiopsis* sp. | *Diaporthe* sp. | *Xylaria* sp. |
| XY006 | 50.0 | 45.5 | 50.0 | 40.0 | 43.8 | 50.3 | - | - | 33.3 |
| XY065 | - | - | - | - | - | - | - | 46.0 | - |
| XY133 | - | - | - | 40.0 | 37.5 | - | - | - | 16.7 |
| XY134 | 40.0 | - | - | - | - | - | - | - | - |
| XY208 | - | - | - | - | - | - | 32.0 | 30.4 | - |
| XY235 | - | - | - | - | 21.4 | - | - | - | - |
| XY236 | - | - | - | - | - | - | - | 30.4 | - |

[a] Determined by measuring the growth inhibition percentage (GI%), which is calculated as [1-(diameter of mycelial growth in the direction of crude extracts/diameter of mycelial growth in the direction of negative control)] ×100%. -, no activity. Only actinomycete isolates showing positive antifungal activities against any of the test fungi are included in the table.

**Supplemental Table S4. IAA production and ACC deaminase activity of endophytic actinomycetes from tea plants**

| Isolate | IAA (μg/mL)[a] | ACCD[b] |
|---|---|---|
| XY006 | 12.7±0.5 | + |
| XY025 | 7.2±2.0 | - |
| XY041 | 4.9±0.3 | - |
| XY042 | 7.4±2.5 | - |
| XY049 | 4.2±0.1 | - |
| XY051 | 43.1±8.7 | - |
| XY065 | 4.3±0.7 | + |
| XY111 | 4.7±0.1 | - |
| XY112 | 6.1±0.2 | - |
| XY133 | ND | - |
| XY134 | 9.5±3.5 | - |
| XY135 | 11.7±0.3 | - |
| XY138 | 8.6±2.0 | - |
| XY139 | 8±0.4 | - |
| XY140 | 6.6±0.2 | - |
| XY141 | 5.9±1.0 | - |
| XY142 | 2.2±0.1 | - |
| XY144 | 8.8±0.3 | - |
| XY145 | 18.7±3.3 | - |
| XY172 | 7.4±0.6 | - |
| XY173 | 6.2±0.1 | - |
| XY174 | 6.3±0.3 | - |
| XY186 | ND | + |
| XY188 | 4.5±0.2 | + |
| XY189 | 4.3±0.2 | - |
| XY190 | 5.4±0.4 | - |
| XY191 | 4.7±0.2 | - |
| XY192 | 8.1±0.2 | - |
| XY199 | 4.8±0.1 | - |
| XY205 | ND | + |
| XY207 | 9.9±0.5 | + |
| XY208 | 7.7±2.0 | - |
| XY209 | 5.1±0.3 | - |
| XY220 | 12.4±0.7 | + |
| XY223 | 9.4±0.8 | - |
| XY224 | 7.2±0.4 | - |
| XY225 | 5.7±0.3 | - |
| XY227 | 6.6±0.1 | - |

| | | |
|---|---|---|
| XY229 | 4.9 ±0.0 | - |
| XY230 | 5.4 ±0.4 | + |
| XY231 | 5.2 ±1.4 | + |
| XY232 | 3.4 ±0.4 | - |
| XY233 | 5.2 ±0.1 | - |
| XY234 | 5.5 ±0.0 | - |
| XY235 | 11.7 ±0.2 | - |
| XY236 | 5.4 ±0.2 | + |

[a] IAA was quantified after growing strains in LB broth in the presence of 5 mM tryptophan. ND, not detected.

[b] ACCD, ACC deaminase; +, present; -, absent.
